# Gingipain proteases from the bacterium *Porphyromonas gingivalis* confer protection against airway viral infection

**DOI:** 10.1073/pnas.2503100123

**Published:** 2026-04-30

**Authors:** Carlos J. Rodriguez Hernandez, Alonso Cruz-Cruz, Chandra L. Shrestha, Marina Terekhova, Phylip Chen, John Perpich, Barbara Potempa, Katherine Carey, Michelle Rohlfing, Mark E. Peeples, Mitchell H. Grayson, Jan Potempa, Maxim Artyomov, Richard J. Lamont, Juhi Bagaitkar

**Affiliations:** ^a^Center for Microbe and Immunity Research, The Abigail Wexner Research Institute at Nationwide Children’s Hospital, Columbus, OH 430205; ^b^Department of Pathology and Immunology, Washington University School of Medicine, Saint Louis, MO 63110; ^c^Department of Oral Immunology and Infectious Diseases, School of Dentistry, University of Louisville, Louisville, KY 40205; ^d^Department of Pharmaceutical Sciences, College of Pharmacy and Health Sciences, Sullivan University, Louisville, KY 40205; ^e^Center for Clinical and Translational Research, The Abigail Wexner Research Institute at Nationwide Children’s Hospital, Columbus, OH 430205; ^f^Division of Allergy and Immunology, Department of Pediatrics, Nationwide Children’s Hospital and The Ohio State University College of Medicine, Columbus, OH 430205; ^g^Department of Pediatrics, The Ohio State University College of Medicine, Columbus, OH 430205

**Keywords:** RSV, periodontitits, interferon, *P. gingivalis*, viral infection

## Abstract

Physical interactions with bacterial colonizers at mucosal barrier surfaces can have beneficial or detrimental effects on viral infectivity and transmission. The oropharyngeal mucosal surface is a major portal of entry for many pathogenic respiratory viruses, such as respiratory syncytial virus (RSV), and also harbors a rich and diverse microbiome. Whether oropharyngeal bacteria directly or indirectly influence host susceptibility to respiratory viruses remains unknown. Here, we show that *Porphyromonas gingivalis*, an oral pathobiont, potently and uniquely suppresses airway epithelial antiviral immunity by degrading interferons (IFNs) and transcriptionally repressing multiple IFN-stimulated genes (ISGs), which are essential for providing resistance to viral infection. Despite inducing a state of IFN hyporesponsiveness, we found that *P. gingivalis* counterintuitively protected against severe infection by RSV and the closely related murine-specific Sendai virus (SeV) in two independent models: human airway bronchial epithelial transwell cultures and airway infection in mice. This protection was conferred by the activity of *P. gingivalis* cysteine proteases (gingipains) that cleaved envelope glycoproteins on RSV and SeV, thereby impairing their infectious capacity. Thus, our data show a nuanced role for *P. gingivalis* in modulating host susceptibility to viral infection. While *P. gingivalis* can significantly inhibit host IFN responses, its proteases preemptively reduce viral infectious capacity, protecting the host from severe damage associated with respiratory infections.

## Significance

Reciprocal interactions between microbial colonizers and host epithelial cells are critical for providing initial defense against viral infections. Here, we present a mechanism that adds another layer of complexity, where proteases produced by host-resident bacteria can directly reduce the infectious capacity of invading viruses, thereby protecting the host from severe viral infection. Specifically, we found that the infectious capacity of the respiratory syncytial virus (RSV) was significantly inhibited upon contact with the proteases (gingipains) produced by the oropharyngeal colonizer *Porphyromonas gingivalis*, rendering them inactive. This preemptive reduction in viral infectious capacity consequently diminished the severity of (RSV) in an interferon-independent manner.

Respiratory syncytial virus (RSV), a member of the *Pneumoviridae* family, is a significant human pathogen associated with considerable morbidity and mortality in infants and the elderly. It is the second leading cause of lower respiratory tract infections in children, with about 3% of those under the age of two requiring hospitalization (1), and also poses a significant health threat in the elderly (2, 3). Despite the high incidence of infection and the development of RSV vaccines, immunity seems to wane over the lifetime, and RSV infections still bear a high risk in susceptible individuals (4). Therefore, understanding the risk factors that influence host susceptibility to RSV infections is crucial for preventing severe illness.

The host microbiome plays an important role in the training and modulation of host immune responses and thereby impacts host susceptibility to pathogenic viral infections. However, the underlying mechanisms are complex and, depending on the context, can have varied outcomes. For example, in the absence of active infection, microbial metabolites and ligands from commensal microbial colonizers drive the low-level activation of host pattern recognition receptors (PRRs), leading to the steady state induction of type I and type III interferons (IFNs) and downstream receptor mediated expression of various IFN-stimulated genes (ISGs) that preemptively block viral infection and train innate and adaptive immunity (5–7). Another level of regulation is set by the direct physical interactions between bacterial colonizers and invading viruses. The synergistic or antagonistic nature of such encounters can influence the clinical severity of disease. For instance, direct binding of viruses to bacteria has been reported to enhance viral infectivity and multiplicity of infection (MOI), thereby driving pathogenesis and adversely impacting the host. Conversely, antagonistic interactions have also been described, in which adsorption on the bacterial surface can disrupt the viral envelope, thereby limiting viral infectivity and preventing infection (8). Most of our understanding of bacterial–viral interactions and their impact on viral pathogenesis has come from studies of enteric viruses. It is still unclear if similar or different interactions influence the infectivity of respiratory viruses.

In humans, the upper airways, throat, and nasopharyngeal passages are heavily colonized by bacteria often associated with oral biofilms (9–11). This is not surprising as the anatomical location of the oral cavity facilitates efficient translocation of oral microbes and respiratory viruses to the oro-respiratory and oro-pharyngeal mucosal surfaces through swallowing, coughing, or microaspiration (12–14). Several studies now show that even in healthy individuals, the lung microbiome is dominated by immigrating oral bacteria (*Prevotella* sp., *Fusobacterium* sp., *Streptococcus* sp., *Corynebacterium* sp., and *Porphyromonas* sp.) that colonize the anatomically contiguous upper and lower airways via subclinical microaspiration (15–17). The presence of *Porphyromonas* sp. in the human airways (16, 18, 19), specifically *Porphyromonas gingivalis* (*Pg*) (20–24), drove our interest, given its role in modulating epithelial antiviral immunity (25).

*P. gingivalis* (*Pg*) is a significant periodontal pathogen that typically resides in the highly anaerobic subgingival niche. It produces numerous virulence factors, including cysteine proteases known as gingipains, which cause broad immune dysregulation by cleaving various cytokines, cellular receptors, extracellular matrix proteins, antimicrobial peptides, and IFN-regulated genes (26, 27). We recently showed that *Pg* is particularly adept at inhibiting oral epithelial IFN responses, using a multihit virulence strategy (25). Given the presence of oral microbes in the human upper airways, including *Pg* (16, 18, 19), we investigated whether *Pg* or *Pg*-derived virulence factors modulate host susceptibility to respiratory viruses such as RSV. Concordant with our observations in the gingival epithelium, *Pg* suppressed IFNs and ISGs but paradoxically protected against viral infection. This protection was imparted by *Pg*-derived proteases, known as gingipains, which rapidly inactivated the infectious capacity of RSV and closely related Sendai virus (SeV) particles, and mitigated the severity of infection in vivo. We find that invasive colonization of the airways by *Pg* was not necessary for viral inactivation, and the introduction of gingipains alone reduced viral loads and protected against virus-driven lung damage. Thus, *Pg*, through the activity of its proteases, creates a bottleneck for viral infection by inactivating viruses, thereby attenuating the severity of viral infection even in an IFN-depleted environment, which would otherwise be conducive to viral pathogenesis.

## Results

### *P. gingivalis* Suppresses IFN Responses in Airway Epithelial Cells.

**Fig. 1.**
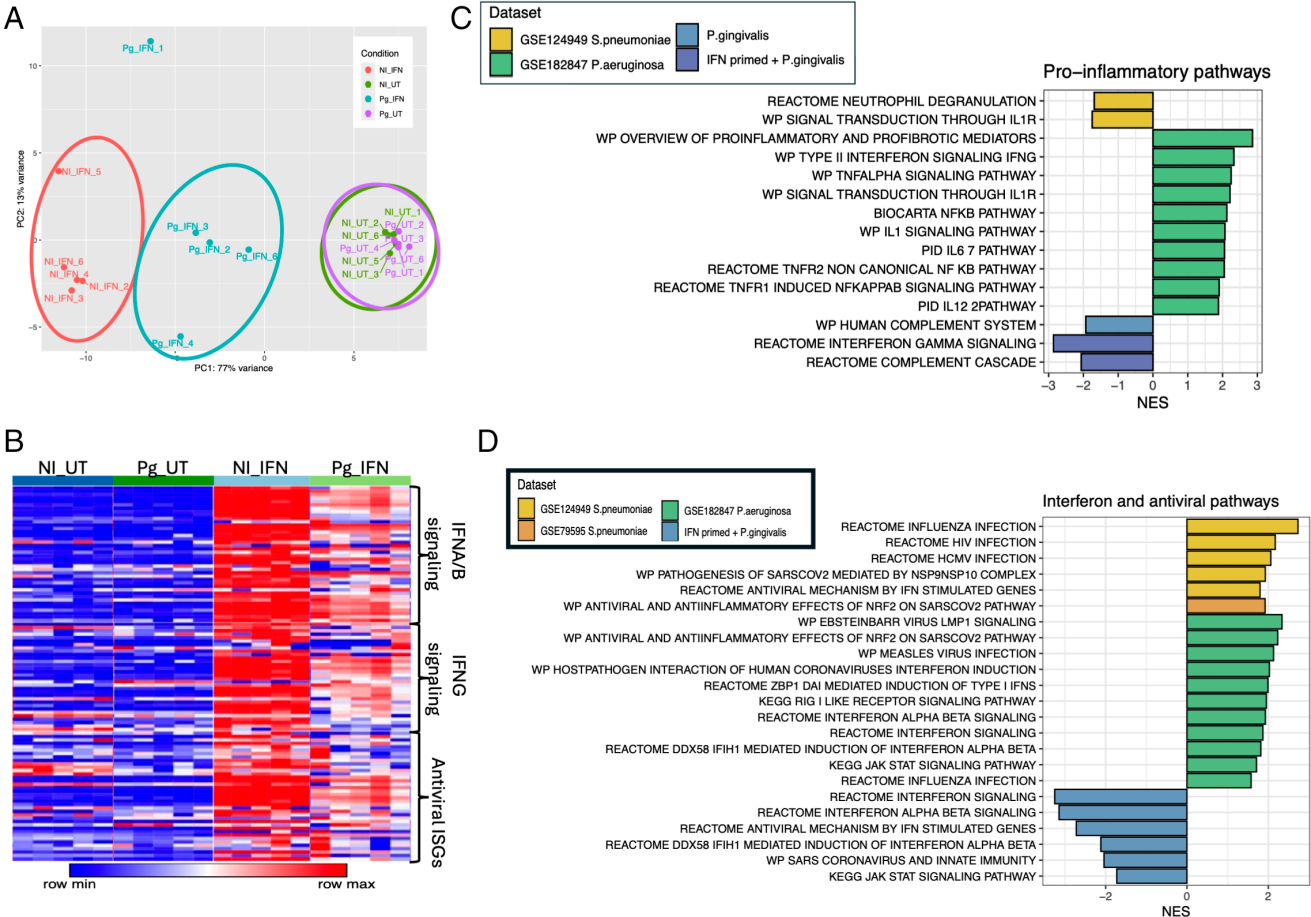
*Pg* infection does not elicit IFN responses in AEC: (*A* and *B*) Naive and IFN-λ primed (20 ng/mL; overnight) A459 cells were infected with *Pg* (MOI 100) for 24 h, and transcriptional responses were analyzed by RNA seq. (*A*) Individual samples were categorized into four groups: untreated cells (NI_UT), IFN-primed cells (NI_IFN), *P. gingivalis*–infected cells (Pg_UT), and IFN-λ primed *Pg* infected cells (Pg_IFN). (*B*) Heatmap, showing log-normalized expression of genes from selected pathways: REACTOME_INTERFERON_ALPHA_BETA_SIGNALING (Stable Identifier: R-HSA-909733), REACTOME_INTERFERON_GAMMA_SIGNALING (Stable Identifier: R-HSA-877300), REACTOME_ANTIVIRAL_MECHANISM_BY_IFN_STIMULATED_GENES (Stable Identifier: R-HSA-1169410). (*C*) Proinflammatory or (*D*) IFN/antiviral pathways were generated from publicly available bulk RNA-seq datasets: A549 cell line infected with *Streptococcus pneumoniae* (GSE79595) or *P. aeruginosa* (GSE182847); nasal mucosal cells infected with *S. pneumoniae* (GSE124949).

First, we determined the transcriptional responses of respiratory epithelial cells to *Pg* infection, including the impact on antiviral pathways. Using the human basal alveolar A549 cell line, which has been extensively used to interrogate immune responses to bacterial pathogens commonly associated with respiratory infections (28, 29), we show that *Pg* can adhere and invade A549 cells within an hour of infection (*SI Appendix*, Fig. S1 *A*–*C*). To broadly profile the impact of *Pg* infection on the transcriptional profiles of naïve and IFN-λ-primed A549 cells, we performed RNA-seq. *Pg* infection modestly, yet significantly upregulated multiple genes involved in extracellular matrix remodeling and lung fibrosis in naïve A549 cells (Fig. 1*A* and *SI Appendix*, Fig. S1 *D*–*G*). However, there was minimal impact on genes in inflammatory and IFN-regulated pathways, which typically orchestrate epithelial antimicrobial defense responses and immune cell recruitment (Fig. 1 *A* and *B*). Intratracheal (i.t.) instillation of *Pg* (10^7^ CFU) into mouse lungs minimally impacted body weight (*SI Appendix*, Fig. S2 *A* and *B*), recruitment of immune cells (*SI Appendix*, Fig. S2*C*), and inflammatory cytokine gene expression (*SI Appendix*, Fig. S2*D*). Thus, our in vivo observations paralleled our in vitro observations. This was unsurprising as *Pg* is found in low numbers in the oral cavities of healthy individuals with no apparent signs of periodontitis (30, 31). It has also been found in the tracheal aspirates of healthy individuals (16, 18, 19), confirming that *Pg* presence within lungs is not typically associated with adverse inflammatory outcomes and may not indicate persistent or invasive infection. Next, we determined whether *Pg* impacted airway epithelial responses to a secondary IFN-inducing stimulus.

We previously demonstrated that *Pg* can actively suppress IFN responses elicited by diverse viral and bacterial ligands in oral epithelial cells downstream of multiple PRRs (25). Thus, we determined whether *Pg* inactivated or interfered with the efficacy of antiviral immune responses such as IFN priming and ISG expression in A549 epithelial cells. Interestingly, consistent with our observations in the oral epithelium, *Pg* markedly suppressed IFN signatures in IFN-λ-primed A549 cells, indicating that *Pg,* upon invasion into epithelial cells, can override the stimulatory effects of IFN priming, as evidenced by a significant loss of ISG expression (Fig. 1 *A* and *B*). *Pg-*infected cells were also unresponsive to a secondary challenge with Poly I:C (a viral mimetic) in A549 cells and other airway epithelial cell lines (*SI Appendix*, Fig. S1 *H* and *I*). Thus, the IFN-suppressing ability of *Pg* was not limited to a single cell line.

Next, we determined whether the IFN-suppressive capacity was unique to *Pg* by comparing the transcriptional signatures induced during *Pg* infection with those from publicly available RNA-seq datasets from other pneumonia-causing bacterial pathogens, such as *S. pneumoniae* and *Pseudomonas aeruginosa*. All datasets utilized the A549 cell line, except the *S. pneumoniae* dataset, which was from nasal epithelium harvested from healthy adult volunteers. Our data show that, in stark contrast to *Pg*, *S. pneumoniae and P. aeruginosa* infection of A549 cells drove the expression of multiple proinflammatory signaling pathways and ISGs (Fig. 1 *C* and *D*). Thus, *Pg* was unique in its capacity to strongly suppress IFNs in the airway epithelium.

### *P. gingivalis* Suppresses IFN Responses During RSV Infection.

**Fig. 2.**
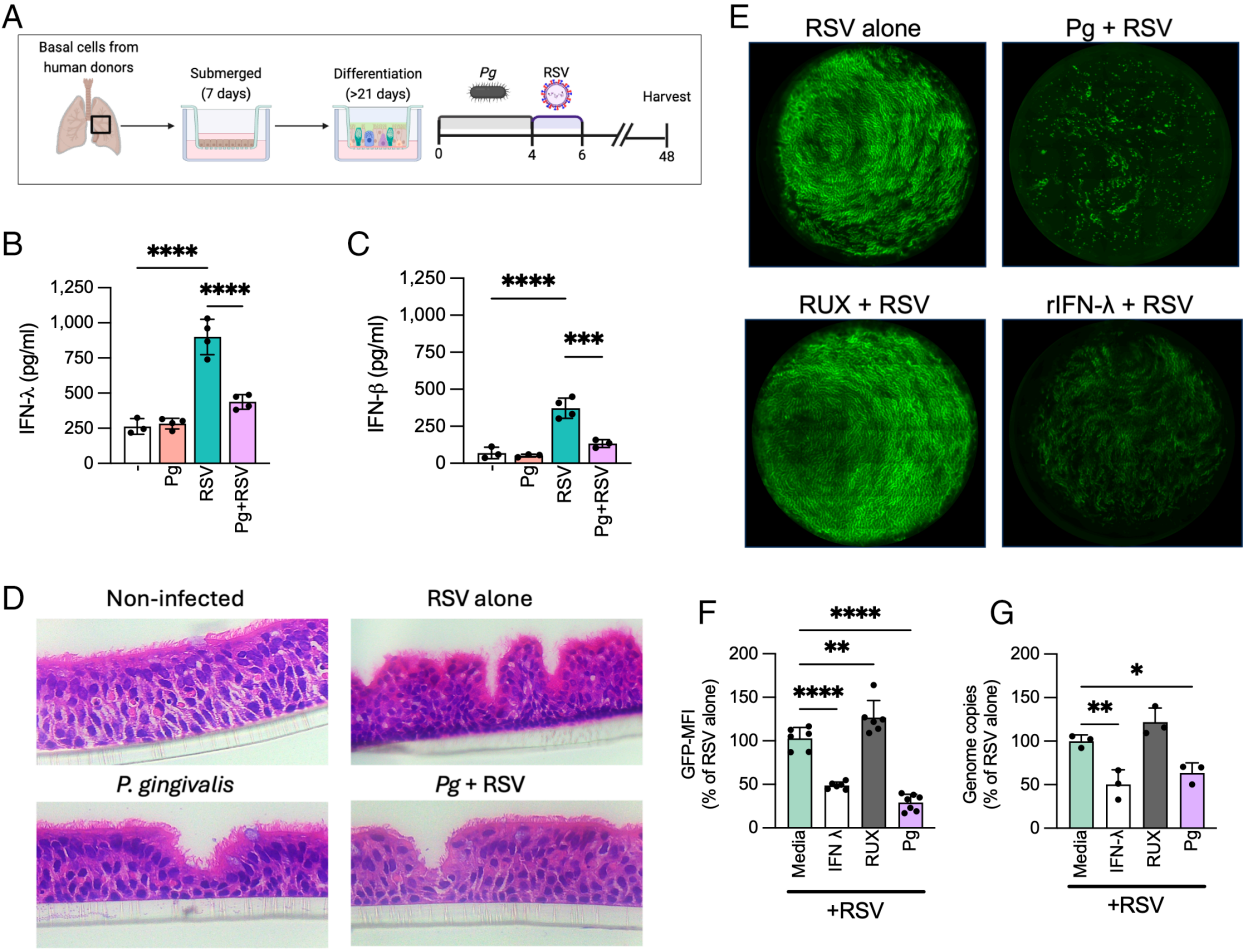
*Pg* restricts IFN-λ responses to RSV infection: (*A*) Graphical depiction of the air–liquid interface human bronchial epithelial (HBE) transwell cultures and differentiation and infection. Transwells were serially infected with *Pg* (0.5 × 10^6^ CFU) for 4 h and then by the rgRSV224-GFP RSV strain (~1,300 PFU) for 2 h and incubated at 37 °C for 48 h. (*B*) IFN-λ and (*C*) IFN-β levels 48 h post–RSV infection are shown as mean ± SD. The Shapiro–Wilk test was used to confirm normal distribution, and data were analyzed using two-way ANOVA (****P < 0.0001). (*D*) Representative H&E images of HBE membranes 48 h past coinfection. HBE transwells were incubated with either 20 ng/ml IFN-λ for 24 h or 10 µM ruxolitinib (RUX) 2 h before *Pg*-RSV infection as described in *A*. Representative images of RSV-infected HBE transwells showing fluorescent infected cells 48 h postinfection are shown in (*E*), and the average GFP-MFI from 3 to 6 wells per group is shown as mean ± SD in (*F*). (*G*) Average genome copies of RSV enumerated by qPCR 48 h postinfection and shown as mean ± SD. For *E* and *G*, the Shapiro–Wilk test was used to confirm normal distribution, and data were analyzed using one-way ANOVA with Tukey post hoc correction (*P < 0.05; **P < 0.01; ****P < 0.0001). Representative data from 3 to 7 individual HBE cultures derived from individual human donors are shown.

Next, we determined whether *Pg*-induced IFN suppression increased susceptibility to respiratory viral infections, such as RSV. To model *Pg*-RSV coinfection, we used a model of air–liquid interface transwell cultures containing human bronchial epithelial cells (HBE) generated by the expansion of bronchial basal cells extracted from human lungs (Fig. 2*A*) (32). Well-differentiated HBE cultures contain ciliated epithelial cells, basal cells, and goblet cells that naturally produce mucus [see H&E and PAS staining (*SI Appendix*, Fig. S3 *A* and *B*)] and thus represent the physiological microenvironment of the human lung. HBE transwells were infected with the rgRSV224 strain (derived from the RSV A2 strain), containing an inserted green fluorescence protein (GFP) gene before the first gene in the full-length cDNA copy of the RSV genome (33). GFP fluorescence thus serves as a “tracer” for quantitatively measuring RSV infection and replication. RSV infection led to a robust IFN-λ production (Fig. 2*B*) and, to a lesser extent, IFN-β (Fig. 2*C*); however, both were significantly suppressed in the presence of *Pg*. We were unable to detect IFN-α or IFN-γ by ELISA. Our observations were not limited to the ATCC 33277 strain, which is fimbriated and considered more invasive; they were also true upon challenge with the encapsulated and nonfimbriated W83 strain of *Pg*. W83 infection reduced IFN levels in A549 epithelial cells (*SI Appendix*, Fig. S3*C*) and in HBE cultures and limited the infective capacity of RSV (*SI Appendix*, Fig. S3 *D* and *E*). Interestingly, the trypsin-like activity of gingipains had minimal impact on the integrity of HBE cellular layers in *Pg* coinfected wells (Fig. 2*D*). Vacuolation and stress responses in HBE layers, typical of RSV infection, were more evident only in RSV-infected wells.

RSV is an IFN-restricted virus, and several IFN-induced antiviral restriction factors, such as the IFN-induced transmembrane proteins (IFITM) 1 and 3, inhibit RSV entry and replication. Their deletion in mice was associated with significant disease and weight loss (34, 35). Other IFN-induced proteins (IFI) 44 and 44L have also been shown to restrict RSV replication and transcription (36). Consistent with this literature, we observed that pretreatment of HBE cultures with rIFN-λ significantly blocked RSV replication. In contrast, pretreatment with ruxolitinib (RUX), a JAK1/2 inhibitor that blocks IFN receptor signaling and downstream ISG expression, augmented RSV replication (Fig. 2 *E* and *F*). Since *Pg* infection creates an IFN-depleted microenvironment, we expected to see a significant increase in RSV replication in *Pg* coinfected cells. However, contrary to our expectation, RSV replication, as indicated by GFP-MFI and genome copy numbers respectively (Fig. 2 *F* and *G*), was significantly dampened in the presence of *Pg*.

### Gingipains Cleave IFN-λ and Strip RSV Glycoproteins, Reducing Viral Infectious Capacity.

*Pg* produces three different endoproteases with trypsin-like activity, known as gingipains. Arginine gingipains (HRgpA and RgpB) and lysine gingipains (Kgp) cleave after the (Arg-X-aa) and Lys-X-aa peptide bonds, respectively (37, 38). Gingipain activity is a crucial aspect of *Pg* pathogenesis, as it can cleave multiple cellular proteins, receptors, and immune factors. We previously demonstrated that all three gingipains drive the proteolysis of the type I and III IFN receptors, thereby blocking downstream ISG activation in the oral epithelium (25). However, whether gingipains are essential in limiting the efficacy of IFN signaling during viral infections in the airway epithelium remains unknown. To determine this, we carried out RSV-*Pg* cochallenge using the ATCC 33277 wildtype (WT), or a triple deletion mutant lacking HRgpA, RgpB, and Kgp (*Δrgpa, Δrgpb, Δkgp*; abbreviated as *ΔKRAB*) (39). We also used the invasion-deficient *ΔfimA* mutant that lacks the major fimbriae essential for binding and invasion into epithelial cells but has intact gingipain activity as an additional control (40). Our data show that purified gingipains proteolytically fragmented IFN-λ (*SI Appendix*, Fig. S3*F*). RSV-induced IFN-λ responses in HBE cultures were also subdued in the presence of the WT *Pg* strain and the *ΔfimA* strain (*SI Appendix*, Fig. S4*A*) and consequently suppressed the downstream induction of multiple antiviral genes (*SI Appendix*, Fig. S4*B*). However, this was not observed during infection with *ΔKRAB.* Gingipains are necessary for processing other surface antigens of *Pg,* and their deletion might impact other virulence factors (41). To circumvent this limitation, we inhibited gingipain activity in the WT strain and observed a similar suppression of IFNs as seen with the *ΔKRAB* mutant (*SI Appendix*, Fig. S4*C*).

**Fig. 3.**
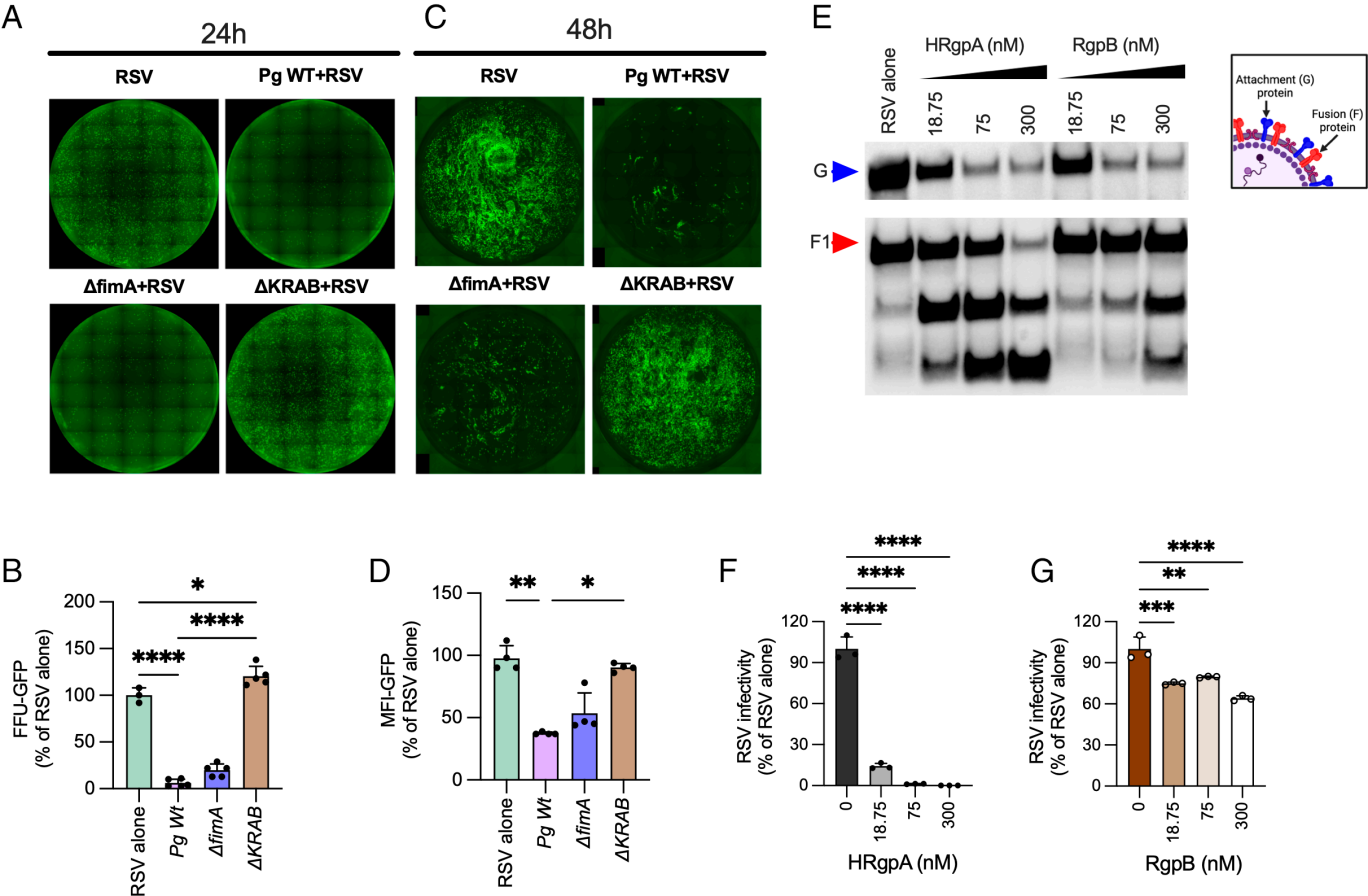
Gingipains impair RSV infectivity: HBE cultures were infected with 0.5 × 10^6^ CFU of wildtype (WT) *Pg* ATCC 33277 or isogenic mutant strain lacking the major fimbrial antigen A(*ΔfimA*); and the triple deletion mutant lacking HRgpA (*ΔrgpA*), RgpB (*ΔrgpB*), and Kgp (*Δkgp*) (abbreviated as *ΔKRAB*) for 4 h. This was followed by a 2 h infection with RSV (~1,300 PFU). (*A*–*D*) Representative images of RSV infection at 24 and 48 h postinfection are shown in *A* and *C*, respectively and corresponding average FFU or MFI from 3 to 4 wells per group is shown as mean ± SD in *B* and *D*. (*E*) 1 × 10^6^ RSV particles were treated with arginine gingipains HRgpA and RgpB at indicated concentrations for 30 min, and RSV attachment (*G*; blue arrow) and fusion (*F*; red arrow) protein integrity (laddering) were determined by a western blot. (*F* and *G*) Hep2 cells were infected with gingipain-treated RSV particles, and infection was determined by quantifying GFP fluorescence 24 h postinfection. Data were normalized to RSV alone (no gingipain treatment) and are shown as mean ± SD. Statistical differences, after normality testing (Shapiro–Wilk test), were measured using one-way ANOVA with Tukey post hoc test for parametric distribution or Kruskal–Wallis post hoc test for nonparametric distribution (*P < 0.05; ***P < 0.001; ****P < 0.0001).

Next, we investigated whether gingipains, independent of their ability to suppress IFNs, also impact RSV infectivity. Respiratory viruses are particularly adept at co-opting host proteases for entry into host cells, virion maturation, and modulation of the host response during infection (42). However, to the best of our knowledge, the impact of bacterial proteases on regulating virion infectivity has not been described. In the HBE model, focus-forming units (FFUs) correlate with the number of infection foci created upon virion binding or the total number of infected cells, as determined by fluorescently labeled viral antigens for nonlytic viruses. In the RSV model, GFP FFUs at 24 h indicate the number of infectious viral particles. At later stages (48 h postinfection), GFP mean fluorescence intensity (MFI) increases due to viral replication and syncytia formation due to ciliary beat characteristic of HBE cultures (43). We observed a significant drop in RSV infectivity (FFUs) in cells coinfected with *Pg* WT at 24 h postinfection but not with the *ΔKRAB* mutant, indicating that *Pg* inhibited viral adsorption or infectivity to airway epithelial cells in a gingipain-dependent manner (Fig. 3 *A* and *B*). This initial reduction in infectious load correlated with a reduction in RSV-GFP MFI at 48 h (Fig. 3 *C* and *D*). The invasion-deficient *ΔfimA* mutant, which lacks the major fimbrial antigen FimA but has intact gingipain activity, showed a similar phenotype to the wild-type strain of *Pg*.

RSV encodes a glycoprotein (G) known as an attachment protein and a fusion (F) protein that play important roles in host cell entry. Unlike in cell lines, where RSV entry relies on G protein binding to cell surface glycosaminoglycans (GAGs), particularly heparin sulfates, infection in HBE cultures occurs exclusively through binding to ciliated cells in a CX3CR1-dependent manner (44, 45). Since eliminating gingipains restored RSV infectivity in *Pg* coinfected cells, we determined whether gingipains incapacitated virions by cleaving RSV envelope glycoproteins. Preincubation of RSV with purified Arg gingipains led to the proteolysis of RSV attachment (G) and fusion (F) proteins (Fig. 3*E*), thereby reducing their infectious capacity in HEp2 (Fig. 3 *F* and *G*).

### *P. gingivalis* Attenuates Viral Loads In Vivo in a Gingipain-Dependent Manner.

Next, we determined whether gingipain-mediated viral inactivation affected the severity of respiratory viral infections in vivo. RSV is a human-adapted virus that infects mice (C57BL/6J strain) poorly at physiological doses (46, 47). Thus, we used SeV, a negative-sense single-stranded RNA virus belonging to the paramyxoviridae family of viruses, which specifically infects rodents. SeV infection in mice parallels the clinical symptomology associated with RSV infection in humans (48). Interestingly, as with RSV, gingipain (Arg-gingipain and Lys-gingipain) mediated proteolysis of the hemagglutinin-neuraminidase (HN) and fusion (F) proteins, both of which are essential for SeV infection (*SI Appendix*, Fig. S5*A*).

**Fig. 4.**
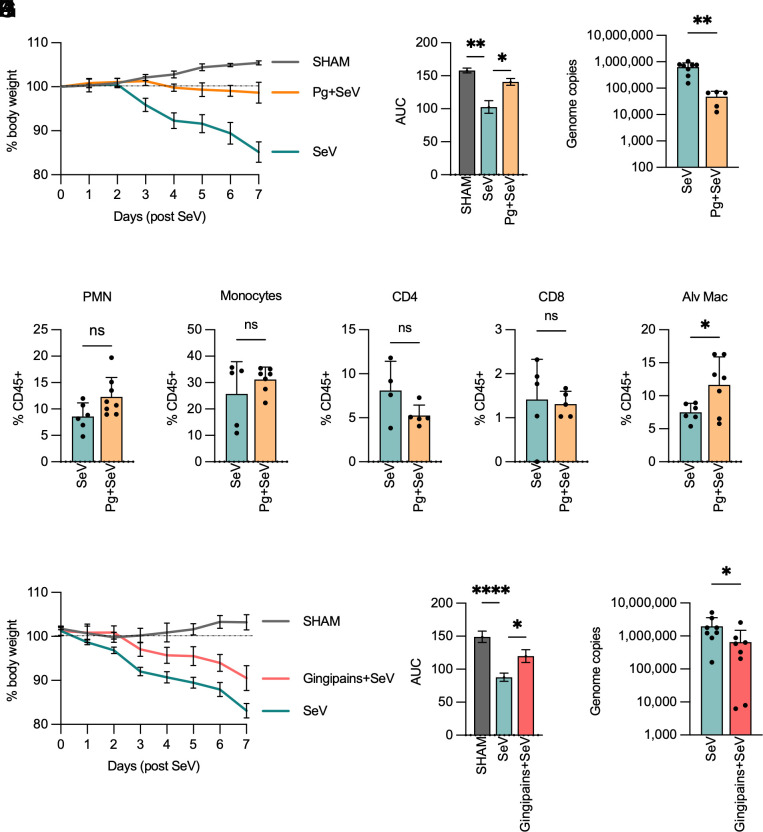
*Pg* and gingipains attenuate SeV infection in vivo: Wildtype mice (5 to 8 per group) were colonized with 10^7^ CFU *Pg* intratracheally and 24 h later challenged intranasally with 2 × 10^5^ PFU SeV. (*A*) Weight loss over 7 d is shown as % of initial body weight and (*B*) area under the curve (AUC) as mean ± SD. Statistical differences, after normality testing (Shapiro–Wilk test), were measured using one-way ANOVA with Tukey post hoc test for parametric distribution. (*C*) SeV genome copies (mean ± SD) in the lung homogenates of mice on Day 7. (*D–H*) Immune cell infiltration in mouse lungs (4 to 5 mice) was determined by flow cytometry, and population abundance is expressed as the percentage of CD45+ cells (mean ± SD). Statistical differences, after normality testing (Shapiro–Wilk test), were measured using an unpaired *t* test (*P < 0.05; **P < 0.01). Gingipains were administered intratracheally to wildtype mice (8 per group) 30 to 60 min prior to intranasal challenge with 2 × 10^5^ PFU SeV. (*I*) Weight loss over 7 d is shown as % of initial body weight and (*J*) AUC as mean ± SD. Statistical differences, after normality testing (Shapiro–Wilk test), were measured using one-way ANOVA with Tukey post hoc test for parametric distribution. (*K*) Viral genome copies (mean ± SD) in the lung homogenates of mice on Day 7. Statistical differences, after normality testing (Shapiro–Wilk test), were assessed using the Mann–Whitney U test (*P < 0.05; **P < 0.01).

*Pg* is a human-adapted pathogen whose biological niche is within the anaerobic environment of the human subgingival cavity. *Pg* can be found in the oral cavities of humans within the first few days of life and is also detected in periodontally healthy individuals, albeit at a lower frequency compared to patients with periodontitis (30, 31). It can translocate to the upper airways and oropharyngeal regions in humans, possibly via salivary flow or microaspiration (16, 18, 19). In mice, this represents a challenge as they are not naturally colonized by *Pg*. Thus, we used a model in which *Pg* was directly instilled into the airways via intratracheal catheters 24 h prior to SeV infection. This approach was used to minimize variability among infected mice and to allow natural interactions with respiratory viruses as they pass through the airways. As expected, SeV infection led to significant morbidity and weight loss in mice with peak viremia on Day 7 postinfection. Interestingly, SeV-driven weight loss was attenuated in the presence of *Pg* and was accompanied by a substantial decrease in viral genome copies in the lungs of infected mice (Fig. 4 *A*–*C*). SeV-infected mouse lungs showed characteristic diffuse damage to the alveolar epithelium and immune cell infiltration. In contrast, mice coinoculated with *Pg* and SeV had reduced lung damage (*SI Appendix*, Fig. S5*B*). Analysis of lung single-cell suspensions revealed no statistically significant differences in inflammatory cell infiltration (Fig. 4 *D*–*H*; see gating strategy in *SI Appendix*, Fig. S5*C*) in SeV alone vs. *Pg* + SeV cohorts, indicating that the protection conferred upon introduction of *Pg* in mouse airways was unrelated to differential immune cell recruitment but more likely related to the physical interaction of *Pg* with the virus and possibly driven by gingipain-mediated attenuation of SeV infectious capacity. To test this hypothesis, we administered purified gingipains (Rgp and Kgp- see methods) directly into the mouse airways (i.t) and then challenged the mice with SeV. Similar to our observations with *Pg* administration, a single dose of gingipains attenuated disease progression, resulting in significantly less weight loss and lower viremia in mice (Fig. 4 *I*–*K*). Thus, our data show that gingipains are necessary and sufficient to reduce viral load and infectious capacity even in the absence of live bacteria.

### *Pg* Coinfection in Ifnlr^−/−^ Mice Exacerbated SeV-Induced Lung Damage Despite a Reduction in Viral Loads.

In our mouse model, a single intratracheal low dose (10^7^ CFU) of *Pg* into the airways effectively reduced SeV infectivity in vivo (Fig. 4); however, it did not consistently inhibit IFN expression. Repeated i.t. instillation, as well as higher, nonphysiologically relevant doses of *Pg* in the lungs, drive lung vascular leakage and significant damage (39) and therefore are not feasible for testing whether *Pg* at higher doses mediates IFN suppression in mouse lungs. Thus, to overcome this limitation and determine the combined impact of *Pg* colonization and IFN suppression in modulating host responses to respiratory viruses, we used IFN-λ receptor knockout mice (*Ifnlr^−/−^*). IFN-λ has been shown to be essential for restricting initial viral infection and spread without causing the damaging inflammation or immunopathology typically associated with Type I IFN signaling (49, 50).

**Fig. 5.**
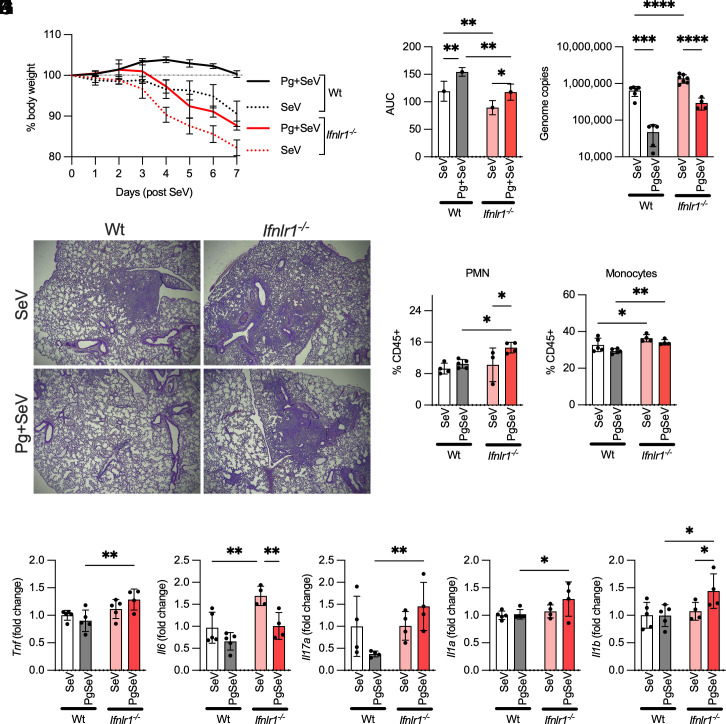
*Pg* infection in *Ifnlr^−/−^* mice is associated with significant lung damage: WT and *Ifnlr^−/−^* mice (N = 5) were colonized with 10^7^ CFU *Pg* intratracheally and 24 h later challenged intranasally with 2 × 10^5^ PFU SeV. (*A*) Weight loss over 7 d is shown as % of initial body weight, and (*B*) AUC as mean ± SD. (*C*) Viral genome copies (mean ± SD) in the lung homogenates of mice on Day 7. (*D*) Representative images of H&E-stained lung tissues on Day 7. (*E* and *F*) Flow staining showing relative % of neutrophils (PMNs) and monocytes in mouse lungs on Day 7. (*G–K*) Inflammatory cytokine transcripts were assessed by qPCR and normalized to GADPH. For *G–K*, data are shown as fold change over the WT-SeV group. Statistical differences, after normality testing (Shapiro–Wilk test), were assessed using Two-way ANOVA (*P < 0.05, **P < 0.01; ****P < 0.001).

We first instilled *Pg* intratracheally into the lungs of WT and *Ifnlr^−/−^*mice, followed by an intranasal SeV challenge 24 h later. As expected, *Ifnlr^−/−^*mice were more susceptible to SeV infection and exhibited a significantly higher weight loss over 7 d with higher viral titers in lung homogenates than the wildtype mice (Fig. 5 *A*–*C*). The presence of *Pg* in mouse lungs still attenuated viral loads and weight loss in *Ifnlr^−/−^*, and WT mice, indicating that *Pg* infection of the airways creates a bottleneck for viral infection, reducing SeV titers independent of IFN-λ signaling (Fig. 5*C*). However, despite the attenuation in viral loads, *Pg* + SeV coinfected *Ifnlr^−/−^*mouse lungs showed significant damage with inflammatory cell (neutrophils and monocytes) recruitment (Fig. 5 *D*–*F*) compared to wildtype mice. Previous studies have shown that IFN-λ signaling during viral infection is essential for regulating neutrophil effector functions and tissue damage (51). Consistent with this, we observed significantly higher neutrophil and monocyte infiltration into the lungs of *Ifnlr^−/−^*mice than in WT mice, which correlated with lung pathology in SeV-infected mice (Fig. 5 *D*–*F*). Interestingly, the introduction of *Pg* into the airways almost entirely blocked inflammatory damage in the lungs of wildtype mice but not *Ifnlr^−/−^*mice (Fig. 5*D*) despite the reduction in viral load (Fig. 5*C*). qPCR analysis of lung homogenates also showed significantly higher levels of multiple proinflammatory cytokine transcripts (Fig. 5 *G*–*K*), consistent with immune cell infiltration and damage. Overall, these data show that the presence of *Pg* in the lungs of immunocompromised mice can reduce viral loads but also contribute to greater damage during respiratory viral infection.

## Discussion

Viruses and bacteria can occupy the same niche within the host, and much remains unknown regarding the molecular nature of viral–bacterial interactions and the collective impact on host susceptibility to viral infection. Our work demonstrates a mechanism by which proteases produced by the oral colonizing bacterium *P. gingivalis* can proteolytically cleave and disable respiratory viruses, thereby decreasing their ability to cause infection. This form of viral–bacterial antagonism is distinct from previously described mechanisms and introduces a new level of complexity to the microbiome’s role in modulating host susceptibility to viral infections, independent of IFN signaling.

Most of our understanding of direct physical interactions between bacteria and viruses emanates from studies of enteric viruses that have to bypass a significant biomass of intestinal bacteria before gaining access to underlying mammalian cells, their primary replicative niche (8). Both synergistic and antagonistic outcomes have been described. For example, binding of the capsid-1 protein from enteric poliovirus to bacterial cell wall components, such as lipopolysaccharides or peptidoglycans, has been shown to enhance the stability of poliovirus particles and enhance their adherence to epithelial cells, thereby promoting infectivity (52, 53). The adsorption of enteric viruses (poliovirus, norovirus) to bacterial adhesins forms multivalent viral–bacterial complexes, which, upon phagocytic uptake, further enhance viral transmission and MOI (53, 54). These examples suggest that certain bacterial species in the gut microbiome play a more “proviral” role by enhancing host susceptibility to viral infection (52, 55). Interestingly, antagonistic interactions can also occur. For example, *Lactobacillus reuteri,* a commensal bacterium, inhibits viral entry into host cells by binding and sequestering the virus away from host cells, thereby limiting infection (56). Very little is known about viral–bacterial interactions at other mucosal surfaces.

The airways are an oxygen-rich environment, which may not be conducive to the survival of an obligate anaerobe such as *Pg*, typically found in the subgingival niche. Our studies show that the ability of *Pg* to limit viral infections almost entirely relies on the activity of gingipains. These observations are based on our in vitro studies using highly specific chemical inhibitors to block gingipain activity in live bacteria, as well as on the use of triple gingipain knockout isogenic strains. Since we were unable to complement this strain, there is a slight possibility of pleiotropic effects that could impact other bacterial traits, confounding our observations. However, these concerns were mitigated to some extent by in vivo experiments where direct instillation of a cocktail of all three gingipains (HRgpA, RgpB, and Kgp) directly into mouse lungs protected from viral pathogenesis and reduced viral loads even in the absence of live bacteria. This approach and data are biologically relevant for several reasons. In patients with periodontitis, gingipain concentrations have been shown to be as high as ~1.5 μM in gingival crevicular fluids (57), and their catalytic activity is not adversely affected in oxygenated environments such as saliva (58, 59). In our hands, low doses ranging 300 nM blocked RSV and SeV infectious capacity. Furthermore, gingipains are actively secreted by *Pg* and are found in extraoral tissues (60). Thus, *Pg,* through the activity of gingipains, may neutralize respiratory viruses at sites more proximal to the oral cavity without directly translocating to the lungs, as indicated in a few recent reports. Our animal model of cochallenge with SeV and *Pg* confirms this modality, as we do not see prolonged colonization or infection of the lung by *Pg*. In our studies, repeated intratracheal inoculations of *Pg* were not possible as this would have led to significant tissue damage, thus we are unable to confirm whether viral neutralization occurs in the lung directly or within the upper airways proximal to the oral cavity.

Our data show that, in addition to aggregation and adsorption, bacterial proteases can affect viral infectivity, and our findings with *Pg* and gingipains align with other reports in the literature. For example, serine proteases from *Bacillaceae* sp. cleave inactivate noroviruses (61), whereas cleavage of the influenza A virus (IAV) hemagglutinin by *P. aeruginosa* and *Staphylococcus aureus* facilitates IAV replication and pathogenicity (62). Interestingly, a recent report by Kamio et al. indicated that gingipains might also aid IAV infectivity in a Madin-Darby canine kidney (MDCK) cell line via hemagglutinin cleavage (63). While we have not directly examined IAV, the effects of gingipains may be virus-specific. However, the model and cell lines used by Kamio et al. differ significantly from those used in our approach. While their work examines the impact of gingipains on the release of IAV from preinfected cells, our work focuses on primary infectivity. Furthermore, interferon suppression induced by *Pg* in epithelial cells may also be a significant contributing factor to viral replication once it overcomes the initial bottleneck and invades epithelial cells.

Our work now consistently and convincingly shows that *Pg* can inhibit IFN-λ in the oral keratinocytes (25), gingival epithelial cells (25), as well as human airway epithelium (Fig. 1). Independent of its role in antiviral immunity, IFN-λ signaling also impacts tissue repair following viral infection. Two recent reports show that IFN-λ signaling in the lungs during high-dose IAV infection or repeated Poly I:C instillation counterintuitively hampered lung repair by inhibiting epithelial proliferation and differentiation (64, 65). Lowered expression of IFN-λ during *Pg* infection might lead to a more fine-tuned or regulated inflammatory response that protects barrier integrity during RSV/SeV coinfection, reducing damage. Interestingly, low-dose instillation of *Pg* in mouse lungs did not induce inflammation and was effective in reducing viral titers in vivo and in protecting against lung damage. Thus, our studies have identified a role for an oral bacterial colonizer in protection against RSV infection and tissue damage.

## Materials and Methods

### Bacteria.

*P. gingivalis* ATCC 33277 (wild type) and W83 strains were cultured in trypticase soy broth (TSB) supplemented with 5 μg/mL hemin, 1 μg/mL menadione, and 1 mg/mL yeast extract. Isogenic mutants on ATCC 33277 background were cultured with selection antibiotics: 10 µg/mL erythromycin for the major fimbriae mutant (*ΔfimA*)(66); 10 μg/mL erythromycin, 0.7 μg/mL tetracycline, and 20 μg/mL chloramphenicol for the triple gingipain mutant *Δkgp ΔrgpA ΔrgpB* abbreviated as (*ΔKRAB*) (39, 67). HaloTag *Pg* on ATCC 33277 background (ΔInlJc/TCOW-Halo-InlJ) was made by conjugation of the *ΔInlJ* mutant with the S-17 strain of *Escherichia coli* harboring the pT-COW plasmid containing Halo-InlJ. HaloTag-*Pg* was cultured with 10 µg/mL erythromycin and 0.7 μg/mL tetracycline. All strains were grown anaerobically (85% N_2_, 10% H_2_, and 5% CO_2_) at 37 °C. For certain experiments, *Pg* 33277 was treated with 100 μM of N-alpha-tosyl-L-lysl chloromethyl ketone (TLCK) for 30 min in an anaerobic chamber to inhibit gingipain activity before infection.

### Gingipain Purification.

HRgpA, RgpB, and Kgp were purified from *P. gingivalis* cell-free culture media by acetone precipitation, size-exclusion chromatography using Sephadex G-150 and affinity chromatography as previously described (68). Purity and specificity of all gingipains was confirmed by active site titrations and western blot analysis (69).

### Virus Production and Quantification.

Recombinant GFP-expressing rgRSV224 is based on the A2 laboratory strain of RSV that carries a GFP tag on the leader sequence of the virus (70). RSV stocks were grown in HEp-2 cells in EMEM-10% FBS. GFP-expressing viruses were serially diluted and titrated on HEp-2 cells by inoculating for 2 h at 37 °C and counting GFP foci at 24 hp. RSV genome copies in infected HBE cultures were determined by qPCR using the Power SYBR Green PCR Master Mix (ThermoFIsher) with primers designed to target the nucleocapsid gene. Forward primer: 5′-GGGAGAGGTAGCTCCAGAATA-3′, and reverse primer sequence: 5′-CTCCTAA TCACGGCTGTAAGAC-3′. Copy numbers were determined using a standard curve of Quantitative Genomic RNA from human RSV strain A2 purchased from ATCC. SeV (strain 52) was purchased from ATCC (cat. number: VR-105). SeV genome copies in mice were quantified using SeV standard as previously described (71). SeV qPCR was performed using TaqMan mastermix and the N3F gene SeV-1237F (5′ GGCGGTGGTGCAATTGAG 3′), N3R gene SeV-1300R (5′ CATGAGCTTCTGTTTCTAGGTCGAT 3′), and MGB probe N gene SeV-1257 (5′ AGCTGTAGACAATGCC 3′) probe sets.

### SEV Infection in Mice.

6- to 10-wk-old C57BL/6 mice were purchased from Jackson Laboratories (Bar Harbor, ME). For the *Pg-*SeV challenge, mice were anesthetized and intubated. A sterile catheter was used for the intratracheal delivery of 10^7^ CFU *Pg* ATCC 33277 suspended in 100 μL of sterile saline. After 24 h, mice were challenged with 2 × 10^5^ PFU of SeV (strain 59, ATCC) intranasally as previously described (48). In certain experiments, mice were administered 0.45 μM gingipains (equal concentrations of RgpA+RgpB+Kgp at a final combined concentration of 0.45 μM) intratracheally and challenged with SeV after 1 h. Body weights were measured daily for 7 d.

### Primary Human Bronchial Epithelial Culture and Differentiation (HBE) and Infection.

HBE progenitors were isolated from human donor airways as previously described (32). Human samples were deidentified prior to use in this study. For setting up HBE transwells, progenitor cells were plated on 0.4 μM pore Corning transwells membranes (6.5 or 12 mm in diameter) and fed with PneumaCult air–liquid interface (ALI) media (Stemcell Technologies) supplemented with ROCK inhibitor in both the apical and basolateral chambers. Media in both chambers was replaced every 2 to 3 d. At 7 d, when the cells were confluent and had formed tight junctions as demonstrated by electrical resistance, the apical medium was removed, and the basal medium was replaced with Pneumacult-ALI Medium (STEMCELL Technologies). The media in the basal chambers was replaced with fresh media every 2 to 3 d, and the apical surface was washed with 100 μL of DMEM to remove accumulating mucus for 4 wk, which is sufficient time for complete differentiation of HBE (43). Fully differentiated HBE transwells (6.5 mm) have approximately 5 × 10^5^ cells. We infected HBE with 0.5 × 10^7^ *Pg* to obtain a MOI of 100 bacteria: 1 epithelial cell. After infection, the apical bacteria-containing fluid was removed and replaced with rgRSV224 inoculum (1300PFU) for 2 h at 37 °C. At the end of this period, the apical fluid was removed, and cultures were incubated at 37 °C. For specific experiments, HBE cultures were treated on the basal side with recombinant 20 ng/mL human IFN-λ1 (Peprotech) or 10 µM Ruxolitinib (Invivogen) before bacterial infection.

### Statistics.

All in vitro experiments were done with technical replicates (triplicates) and repeated 3 to 5 times independently. Data from a representative experiment are shown for most in vitro studies. For in vivo experiments, the number of mice per group is indicated in the legend of each experiment. Statistical analyses were performed using GraphPad Prism 10 (version 10.4.1). First, normality testing was done using the Shapiro–Wilk test, and parametric or nonparametric analyses were done using the appropriate post hoc test. A *P* value <0.05 was considered statistically significant. A detailed description of the statistical tests used is stated in each figure legend.

## Supplementary Material

Appendix 01 (PDF)

Dataset S01 (TXT)

## Data Availability

Data used in the study are available in a persistent repository upon publication (GSE281938) (72). Other previously published datasets used for this work are GSE182847 (73), GSE124949 (74), and GSE79595 (75). Additional methods are listed in supporting information.

## References

[1] J. M. Langley , Incidence of respiratory syncytial virus lower respiratory tract infections during the first 2 years of life: A prospective study across diverse global settings. J. Infect. Dis. **226**, 374–385 (2022). 35668702 10.1093/infdis/jiac227 PMC9417131

[2] A. R. Falsey, E. E. Walsh, Respiratory syncytial virus infection in elderly adults. Drugs Aging **22**, 577–587 (2005). 16038573 10.2165/00002512-200522070-00004 PMC7099998

[3] K. A. Linder, P. N. Malani, RSV infection in older adults. JAMA **330**, 1200–1200 (2023). 37676666 10.1001/jama.2023.16932

[4] S. M. Varga, T. J. Braciale, The adaptive immune response to respiratory syncytial virus. Curr. Top. Microbiol. Immunol. **372**, 155–171 (2013). 24362689 10.1007/978-3-642-38919-1_8

[5] E. S. Winkler, L. B. Thackray, A long-distance relationship: The commensal gut microbiota and systemic viruses. Curr. Opin. Virol. **37**, 44–51 (2019). 31226645 10.1016/j.coviro.2019.05.009 PMC6768733

[6] S. F. Erttmann , The gut microbiota prime systemic antiviral immunity via the cGAS-STING-IFN-I axis. Immunity **55**, 847–861.e810 (2022). 35545033 10.1016/j.immuni.2022.04.006

[7] J. A. Van Winkle , Homeostatic interferon-lambda response to bacterial microbiota stimulates preemptive antiviral defense within discrete pockets of intestinal epithelium. eLife **11**, e74072 (2022). 35137688 10.7554/eLife.74072 PMC8853662

[8] D. E. Campbell, Y. Li, H. Ingle, M. T. Baldridge, Impact of the microbiota on viral infections. Annu. Rev. Virol. **10**, 371–395 (2023). 37071931 10.1146/annurev-virology-111821-115754 PMC10543481

[9] A. R. Sonawane , Microbiome-transcriptome interactions related to severity of respiratory syncytial virus infection. Sci. Rep. **9**, 13824 (2019). 31554845 10.1038/s41598-019-50217-w PMC6761288

[10] S. M. Teo , The infant nasopharyngeal microbiome impacts severity of lower respiratory infection and risk of asthma development. Cell Host Microbe **17**, 704–715 (2015). 25865368 10.1016/j.chom.2015.03.008 PMC4433433

[11] C. Ptaschinski, N. W. Lukacs, Early life respiratory syncytial virus infection and asthmatic responses. Immunol. Allergy Clin. North Am. **39**, 309–319 (2019). 31284922 10.1016/j.iac.2019.03.002

[12] S. Kageyama , High-resolution detection of translocation of oral bacteria to the gut. J. Dent. Res. **102**, 752–758 (2023). 37204134 10.1177/00220345231160747 PMC10288163

[13] R. Shigdel , Oral bacterial composition associated with lung function and lung inflammation in a community-based Norwegian population. Respir. Res. **24**, 183 (2023). 37438766 10.1186/s12931-023-02491-6 PMC10337198

[14] N. Wang, J. Y. Fang, *Fusobacterium nucleatum*, a key pathogenic factor and microbial biomarker for colorectal cancer. Trends Microbiol. **31**, 159–172 (2023). 36058786 10.1016/j.tim.2022.08.010

[15] G. B. Huffnagle, R. P. Dickson, N. W. Lukacs, The respiratory tract microbiome and lung inflammation: A two-way street. Mucosal Immunol. **10**, 299–306 (2017). 27966551 10.1038/mi.2016.108 PMC5765541

[16] C. M. Bassis , Analysis of the upper respiratory tract microbiotas as the source of the lung and gastric microbiotas in healthy individuals. mBio **6**, e00037 (2015). 25736890 10.1128/mBio.00037-15 PMC4358017

[17] A. Venkataraman , Application of a neutral community model to assess structuring of the human lung microbiome. mBio **6**, e02284-14 (2015). 25604788 10.1128/mBio.02284-14 PMC4324308

[18] M. S. Muhlebach , Anaerobic bacteria cultured from cystic fibrosis airways correlate to milder disease: A multisite study. Eur. Respir. J. **52**, 1800242 (2018). 29946004 10.1183/13993003.00242-2018 PMC6376871

[19] M. Y. Lim , Analysis of the association between host genetics, smoking, and sputum microbiota in healthy humans. Sci. Rep. **6**, 23745 (2016). 27030383 10.1038/srep23745 PMC4814871

[20] L. Zhijun, Y. Wenhai, Z. Peibin, L. Qingming, Pediatric pulmonary infection caused by oral obligate anaerobes: Case series. Front. Pediatr. **11**, 1226706 (2023). 37744449 10.3389/fped.2023.1226706 PMC10513053

[21] Y. Liu , Clinical significance and prognostic value of *Porphyromonas gingivalis* infection in lung cancer. Transl. Oncol. **14**, 100972 (2021). 33279803 10.1016/j.tranon.2020.100972 PMC7718477

[22] X. Chen, L. Wu, R. Wu, J. Dong, A pulmonary abscess caused by *Porphyromonas gingivalis* infection: A case report and literature review. Clin. Respir. J. **19**, e70099 (2025). 40667600 10.1111/crj.70099 PMC12264576

[23] Y. Li , Comprehensive human respiratory genome catalogue underlies the high resolution and precision of the respiratory microbiome. Brief Bioinf. **26**, bbae620 (2024). 10.1093/bib/bbae620 PMC11586125 39581874

[24] J. Sha , Pyopneumothorax with bronchopleural fistula due to pulmonary infection caused by *Porphyromonas gingivalis* in a patient with periodontitis. Clin. Respir. J. **17**, 962–965 (2023). 37573789 10.1111/crj.13684 PMC10500317

[25] C. J. Rodriguez-Hernandez , Microbiome-mediated incapacitation of interferon lambda production in the oral mucosa. Proc. Natl. Acad. Sci. U.S.A. **118**, e2105170118 (2021). 34921113 10.1073/pnas.2105170118 PMC8713781

[26] G. Hajishengallis, *Porphyromonas gingivalis*-host interactions: Open war or intelligent guerilla tactics? Microbes Infect. **11**, 637–645 (2009). 19348960 10.1016/j.micinf.2009.03.009 PMC2704251

[27] K. N. Cooper , Limited proteolysis of neutrophil granule proteins by the bacterial protease RgpB depletes neutrophil antimicrobial capacity. J. Leukoc. Biol. **117**, qiae209 (2024). 10.1093/jleuko/qiae209 PMC11878996 39319408

[28] N. Alphonse , A family of conserved bacterial virulence factors dampens interferon responses by blocking calcium signaling. Cell **185**, 2354–2369.e2317 (2022). 35568036 10.1016/j.cell.2022.04.028 PMC9596379

[29] S. S. Perelman , Cell-based screen identifies human interferon-stimulated regulators of *Listeria monocytogenes* infection. PLoS Pathog. **12**, e1006102 (2016). 28002492 10.1371/journal.ppat.1006102 PMC5176324

[30] A. L. Griffen, M. R. Becker, S. R. Lyons, M. L. Moeschberger, E. J. Leys, Prevalence of *Porphyromonas gingivalis* and periodontal health status. J. Clin. Microbiol. **36**, 3239–3242 (1998). 9774572 10.1128/jcm.36.11.3239-3242.1998 PMC105308

[31] D. L. McClellan, A. L. Griffen, E. J. Leys, Age and prevalence of *Porphyromonas gingivalis* in children. J. Clin. Microbiol. **34**, 2017–2019 (1996). 8818903 10.1128/jcm.34.8.2017-2019.1996 PMC229175

[32] M. L. Fulcher, S. Gabriel, K. A. Burns, J. R. Yankaskas, S. H. Randell, Well-differentiated human airway epithelial cell cultures. Methods Mol. Med. **107**, 183–206 (2005). 15492373 10.1385/1-59259-861-7:183

[33] L. K. Hallak, P. L. Collins, W. Knudson, M. E. Peeples, Iduronic acid-containing glycosaminoglycans on target cells are required for efficient respiratory syncytial virus infection. Virology **271**, 264–275 (2000). 10860881 10.1006/viro.2000.0293

[34] S. E. Smith , Interferon-induced transmembrane protein 1 restricts replication of viruses that enter cells via the plasma membrane. J. Virol. **93**, e02003-18 (2019). 30567988 10.1128/JVI.02003-18 PMC6401438

[35] W. Zhang , Human respiratory syncytial virus infection is inhibited by IFN-induced transmembrane proteins. J. Gen. Virol. **96**, 170–182 (2015). 25228491 10.1099/vir.0.066555-0

[36] D. C. Busse , Interferon-induced protein 44 and interferon-induced protein 44-like restrict replication of respiratory syncytial virus. J. Virol. **94**, e00297-20 (2020). 32611756 10.1128/JVI.00297-20 PMC7459546

[37] K. Hocevar, J. Potempa, B. Turk, Host cell-surface proteins as substrates of gingipains, the main proteases of *Porphyromonas gingivalis*. Biol. Chem. **399**, 1353–1361 (2018). 29927743 10.1515/hsz-2018-0215

[38] W. A. Chen, Y. Dou, H. M. Fletcher, D. S. Boskovic, Local and systemic effects of *Porphyromonas gingivalis* infection. Microorganisms **11**, 470 (2023). 36838435 10.3390/microorganisms11020470 PMC9963840

[39] M. Benedyk , Gingipains: Critical factors in the development of aspiration pneumonia caused by *Porphyromonas gingivalis*. J. Innate Immun. **8**, 185–198 (2016). 26613585 10.1159/000441724 PMC4801669

[40] M. N. Sztukowska , *Porphyromonas gingivalis* initiates a mesenchymal-like transition through ZEB1 in gingival epithelial cells. Cell Microbiol. **18**, 844–858 (2016). 26639759 10.1111/cmi.12554 PMC5135094

[41] T. Kadowaki , Arg-gingipain acts as a major processing enzyme for various cell surface proteins in *Porphyromonas gingivalis*. J. Biol. Chem. **273**, 29072–29076 (1998). 9786913 10.1074/jbc.273.44.29072

[42] B. Lubinski, G. R. Whittaker, Host cell proteases involved in human respiratory viral infections and their inhibitors: A review. Viruses **16**, 984 (2024). 38932275 10.3390/v16060984 PMC11209347

[43] T. King, A. Mejias, O. Ramilo, M. E. Peeples, The larger attachment glycoprotein of respiratory syncytial virus produced in primary human bronchial epithelial cultures reduces infectivity for cell lines. PLoS Pathog. **17**, e1009469 (2021). 33831114 10.1371/journal.ppat.1009469 PMC8057581

[44] R. A. Tripp , CX3C chemokine mimicry by respiratory syncytial virus G glycoprotein. Nat. Immunol. **2**, 732–738 (2001). 11477410 10.1038/90675

[45] J. S. McLellan, W. C. Ray, M. E. Peeples, Structure and function of respiratory syncytial virus surface glycoproteins. Curr. Top. Microbiol. Immunol. **372**, 83–104 (2013). 24362685 10.1007/978-3-642-38919-1_4 PMC4211642

[46] R. E. Sacco, R. K. Durbin, J. E. Durbin, Animal models of respiratory syncytial virus infection and disease. Curr. Opin. Virol. **13**, 117–122 (2015). 26176495 10.1016/j.coviro.2015.06.003 PMC4699663

[47] M. J. Altamirano-Lagos , Current animal models for understanding the pathology caused by the Respiratory Syncytial Virus. Front. Microbiol. **10**, 873 (2019). 31130923 10.3389/fmicb.2019.00873 PMC6510261

[48] J. Resiliac, J. Santoro, S. A. Hussain, M. Rohlfing, M. H. Grayson, Mouse model of Sendai virus-induced lung disease. Methods Mol. Biol. **2506**, 57–65 (2022). 35771463 10.1007/978-1-0716-2364-0_4

[49] I. E. Galani , Interferon-lambda mediates non-redundant front-line antiviral protection against influenza virus infection without compromising host fitness. Immunity **46**, 875–890.e876 (2017). 28514692 10.1016/j.immuni.2017.04.025

[50] A. Broggi, F. Granucci, I. Zanoni, Type III interferons: Balancing tissue tolerance and resistance to pathogen invasion. J. Exp. Med. **217**, e20190295 (2020). 31821443 10.1084/jem.20190295 PMC7037241

[51] A. Broggi, Y. Tan, F. Granucci, I. Zanoni, *IFN*-lambda suppresses intestinal inflammation by non-translational regulation of neutrophil function. Nat. Immunol. **18**, 1084–1093 (2017). 28846084 10.1038/ni.3821 PMC5701513

[52] S. K. Kuss , Intestinal microbiota promote enteric virus replication and systemic pathogenesis. Science **334**, 249–252 (2011). 21998395 10.1126/science.1211057 PMC3222156

[53] C. M. Robinson, P. R. Jesudhasan, J. K. Pfeiffer, Bacterial lipopolysaccharide binding enhances virion stability and promotes environmental fitness of an enteric virus. Cell Host Microbe **15**, 36–46 (2014). 24439896 10.1016/j.chom.2013.12.004 PMC3920179

[54] A. K. Erickson , Bacteria facilitate enteric virus co-infection of mammalian cells and promote genetic recombination. Cell Host Microbe **23**, 77–88.e75 (2018). 29290575 10.1016/j.chom.2017.11.007 PMC5764776

[55] M. T. Baldridge , Commensal microbes and interferon-lambda determine persistence of enteric murine norovirus infection. Science **347**, 266–269 (2015). 25431490 10.1126/science.1258025 PMC4409937

[56] L. Y. Ang , Antiviral activity of *Lactobacillus reuteri* Protectis against Coxsackievirus A and Enterovirus 71 infection in human skeletal muscle and colon cell lines. Virol. J. **13**, 111 (2016). 27341804 10.1186/s12985-016-0567-6 PMC4920999

[57] A. Guentsch , Comparison of gingival crevicular fluid sampling methods in patients with severe chronic periodontitis. J. Periodontol. **82**, 1051–1060 (2011). 21235330 10.1902/jop.2011.100565 PMC3129431

[58] S. Park, K. Park, H. S. Na, J. Chung, H. Yang, Washing- and separation-free electrochemical detection of *Porphyromonas gingivalis* in saliva for initial diagnosis of periodontitis. Anal. Chem. **93**, 5644–5650 (2021). 33770438 10.1021/acs.analchem.1c00572

[59] U. K. Gursoy , Elevated baseline salivary protease activity may predict the steadiness of gingival inflammation during periodontal healing: A 12-week follow-up study on adults. Pathogens **9**, 751 (2020). 32942694 10.3390/pathogens9090751 PMC7558121

[60] H. Okamura , Outer membrane vesicles of *Porphyromonas gingivalis*: Novel communication tool and strategy. Jpn. Dent. Sci. Rev. **57**, 138–146 (2021). 34484474 10.1016/j.jdsr.2021.07.003 PMC8399048

[61] S. Yamamoto , Bacillaceae serine proteases and *Streptomyces* epsilon-poly-L-lysine synergistically inactivate Caliciviridae by inhibiting RNA genome release. Sci. Rep. **14**, 15181 (2024). 38956295 10.1038/s41598-024-65963-9 PMC11219925

[62] H. Scheiblauer, M. Reinacher, M. Tashiro, R. Rott, Interactions between bacteria and influenza A virus in the development of influenza pneumonia. J. Infect. Dis. **166**, 783–791 (1992). 1527412 10.1093/infdis/166.4.783

[63] N. Kamio, M. E. Cueno, A. Takagi, K. Imai, *Porphyromonas gingivalis* gingipain potentially activates influenza A virus infectivity through proteolytic cleavage of viral hemagglutinin. J. Biol. Chem. **301**, 108166 (2025). 39793895 10.1016/j.jbc.2025.108166 PMC11834065

[64] A. Broggi , Type III interferons disrupt the lung epithelial barrier upon viral recognition. Science **369**, 706–712 (2020). 32527925 10.1126/science.abc3545 PMC7292499

[65] J. Major , Type I and III interferons disrupt lung epithelial repair during recovery from viral infection. Science **369**, 712–717 (2020). 32527928 10.1126/science.abc2061 PMC7292500

[66] R. M. Love, M. D. McMillan, Y. Park, H. F. Jenkinson, Coinvasion of dentinal tubules by *Porphyromonas gingivalis* and *Streptococcus gordonii* depends upon binding specificity of streptococcal antigen I/II adhesin. Infect. Immun. **68**, 1359–1365 (2000). 10678948 10.1128/iai.68.3.1359-1365.2000 PMC97289

[67] J. Y. Lee , Maturation of the Mfa1 fimbriae in the oral pathogen *Porphyromonas gingivalis*. Front. Cell. Infect. Microbiol. **8**, 137 (2018). 29868494 10.3389/fcimb.2018.00137 PMC5954841

[68] J. Potempa, K. A. Nguyen, Purification and characterization of *gingipains*. Curr. Protoc. Protein Sci. **Chapter 21**, 21.20.1–21.20.27 (2007). 10.1002/0471140864.ps2120s49 18429320

[69] J. Potempa, R. Pike, J. Travis, Titration and mapping of the active site of cysteine proteinases from *Porphyromonas gingivalis* (gingipains) using peptidyl chloromethanes. Biol. Chem. **378**, 223–230 (1997). 9165075 10.1515/bchm.1997.378.3-4.223

[70] S. Kwilas , Respiratory syncytial virus grown in vero cells contains a truncated attachment protein that alters its infectivity and dependence on glycosaminoglycans. J. Virol. **83**, 10710–10718 (2009). 19656891 10.1128/JVI.00986-09 PMC2753119

[71] A. M. Akk , Dipeptidyl peptidase I-dependent neutrophil recruitment modulates the inflammatory response to Sendai virus infection. J. Immunol. **180**, 3535–3542 (2008). 18292580 10.4049/jimmunol.180.5.3535 PMC2597084

[72] C. J. Rodriguez-Hernandez , Porphyromonas gingivalis inhibits Respiratory Syncytial Virus infection while suppressing anti-viral immunity. GEO. https://www.ncbi.nlm.nih.gov/geo/query/acc.cgi?acc=GSE281938. Deposited 14 November 2024.

[73] K. Leskinen , Expression profiling by high throughput sequencing. GEO. https://www.ncbi.nlm.nih.gov/geo/query/acc.cgi?acc=GSE182847. Deposited 26 August 2021.

[74] S. P. Jochems, Expression profiling by high throughput sequencing. GEO. https://www.ncbi.nlm.nih.gov/geo/query/acc.cgi?acc=GSE124949. Deposited 11 January 2019.

[75] R. Aprianto, J. Slager, S. Holsappel, J. Veening, Expression profiling by high throughput sequencing. GEO. https://www.ncbi.nlm.nih.gov/geo/query/acc.cgi?acc=GSE79595. Deposited 24 March 2016.

